# 

**Published:** 1882-10

**Authors:** 


*Vol. XXXVI.* **OCTOBER, 1882.** *No. 10.*

### THE

### Dental Register.

A MONTHLY JOURNAL,

**DEVOTED TO THE INTERESTS OF THE PROFESSION.**

EDITED BY J. TAFT, D.D.S.

PUBLISHED BY

SPENCER & CROCKER,

OHIO DENTAL DEPOT,

Nos. 117, 119, 121 WEST FIFTH STREET, CINCINNATI, OHIO.

**TERMS:—$2.00 Per Annum, in Advance. Single Copies, 25 cts.**

